# Supplementary figures and images for: Common gardens in teosintes reveal the establishment of a syndrome of adaptation to altitude
Source: PLoS Genet. 2019 Dec 20;15(12):e1008512. doi: 10.1371/journal.pgen.1008512 (PMC6944379; doi:10.1371/journal.pgen.1008512)

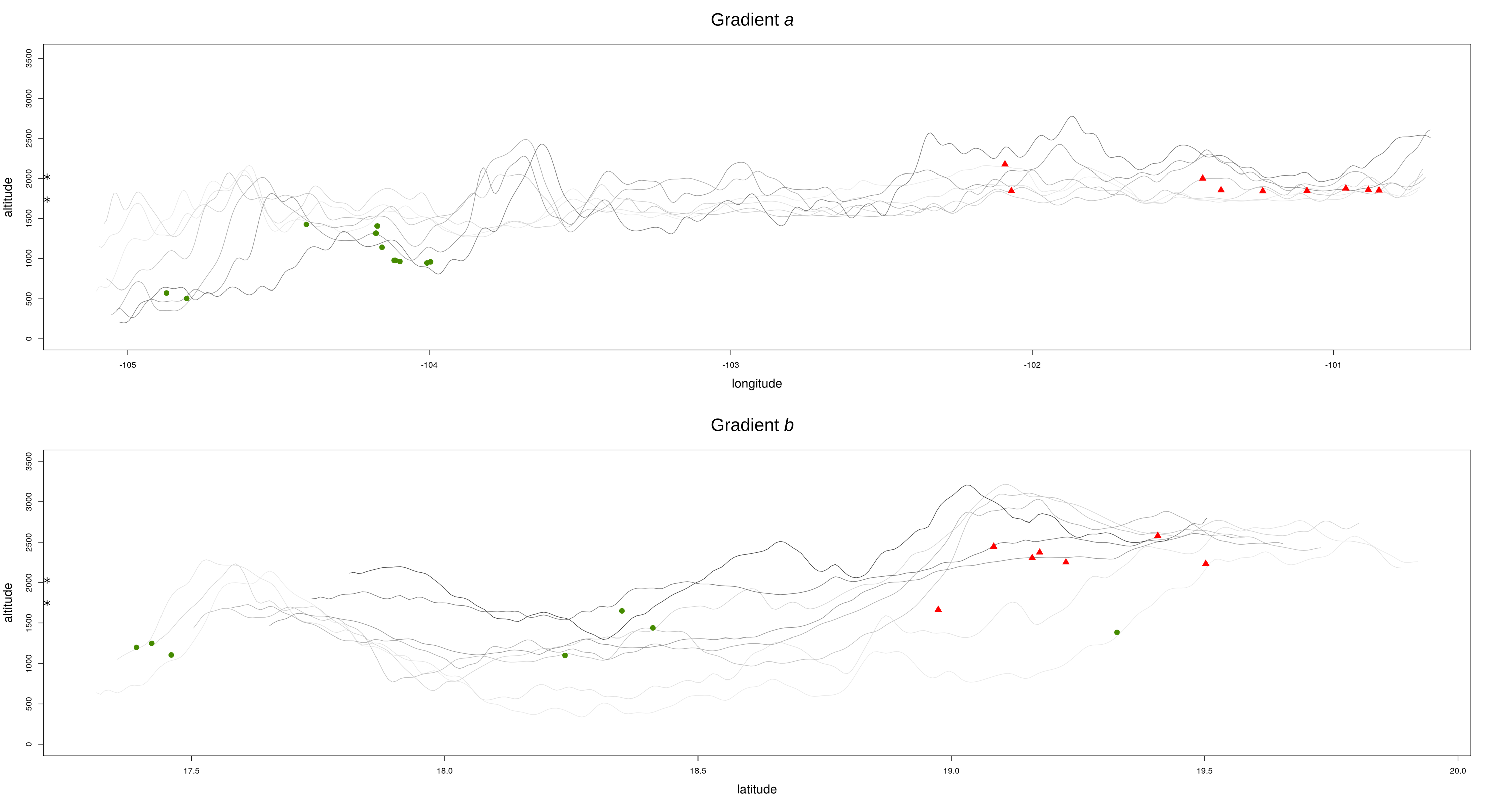

Supplement: S1 Fig — Sampled populations are plotted on parallel altitudinal profiles for gradients a and b. Darker gray lines indicate lower latitude for gradient a and lower longitude for gradient b. Sampled populations are plotted by green circles (parviglumis) or red triangles (mexicana). The altitude of the two experimental fields (CEBAJ: 1750m and SENGUA: 2017m) are marked with asterisks on the y-axes. (TIF) [file pgen.1008512.s001.tif]

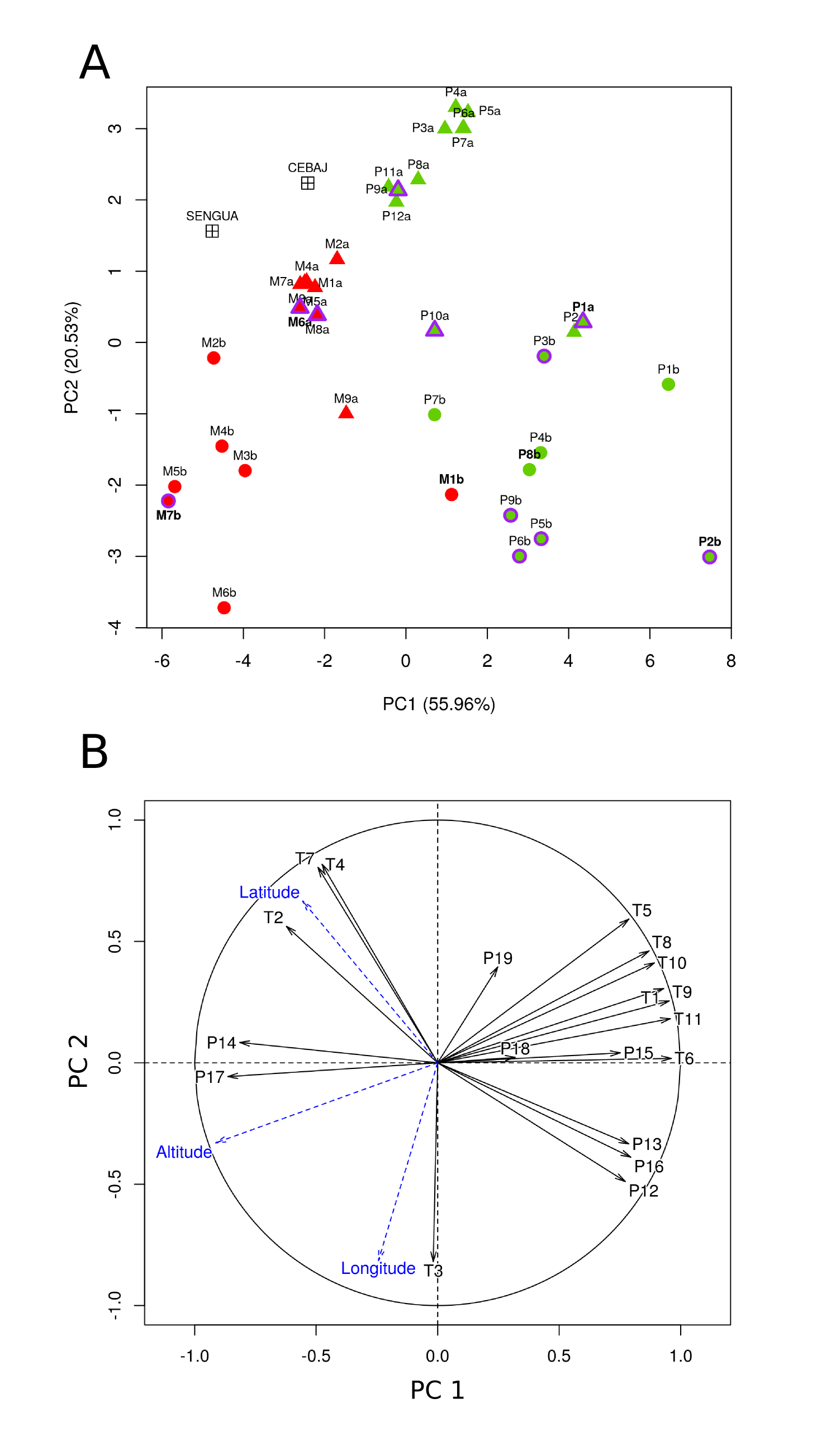

Supplement: S2 Fig — A: Projection of parviglumis (in green) and mexicana (in red) populations on the first PCA plane with gradients a and b indicated by triangles and circles, respectively. The 11 populations evaluated in common gardens are surrounded by a purple outline. Populations that were previously sequenced to detect selection footprints are shown in bold (S1 Table). B: Correlation circle of the 19 climatic variables on the first PCA plane. Climatic variables indicated as Tn (n from 1 to 11) and Pn (n from 12 to 19) are related to temperature and precipitation, respectively. Altitude, Latitude and Longitude (in blue) were added as supplementary variables, and CEBAJ and SENGUA field locations were added as supplementary individuals. (TIF) [file pgen.1008512.s002.tif]

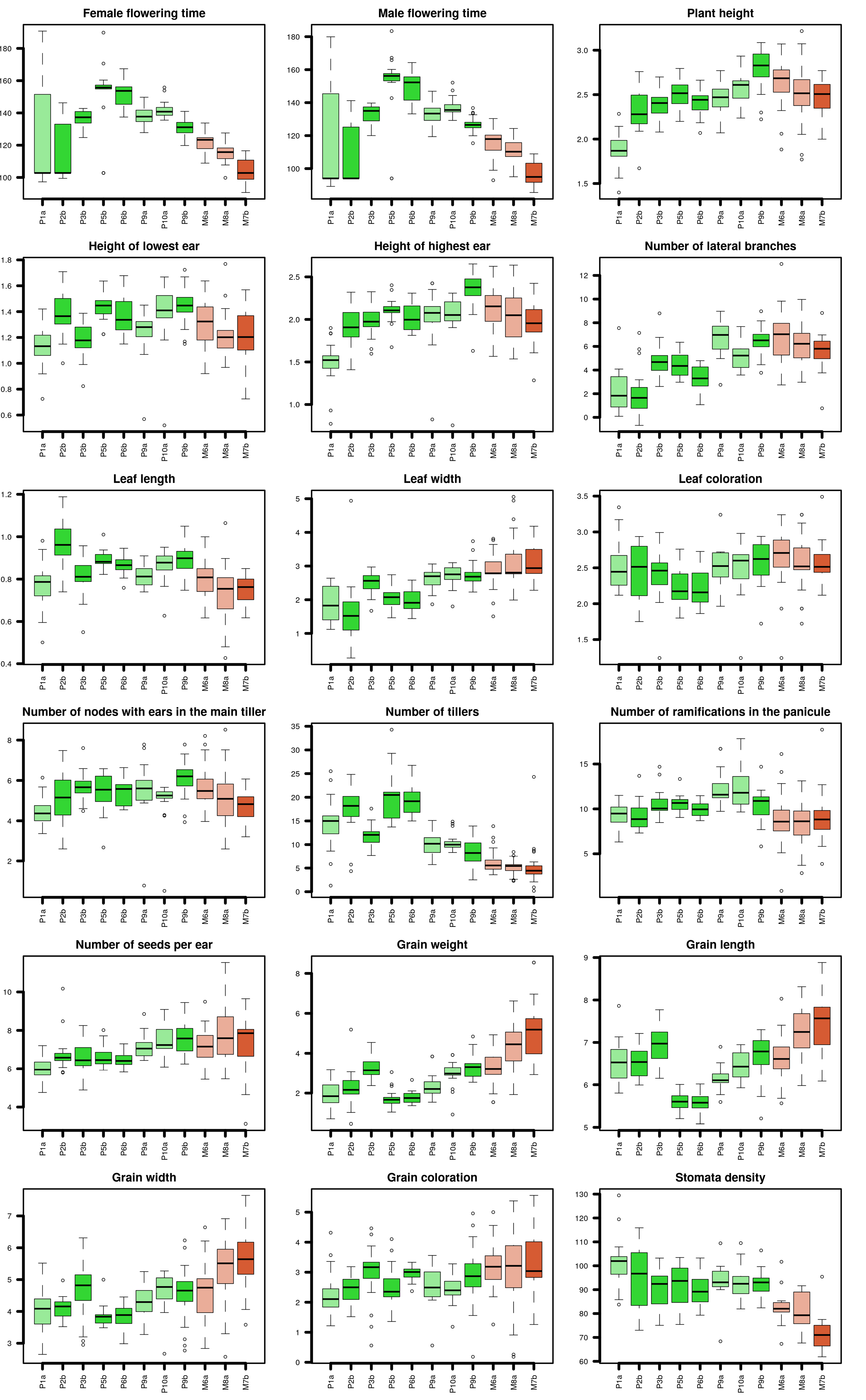

Supplement: S3 Fig — Populations are ranked by altitude. parviglumis populations are shown in green and mexicana in red. Lighter colors are used for gradient ‘a’ and darker colors for gradient ‘b’. Units of measurement correspond to those defined in S2 Table. For male and female flowering time, we report values for all 11 populations although very few individuals from the two most lowland populations (P1a and P2b) flowered. Covariation with altitude was significant for all traits except for the number of nodes with ears on the main tiller (S3 Table). (TIF) [file pgen.1008512.s003.tif]

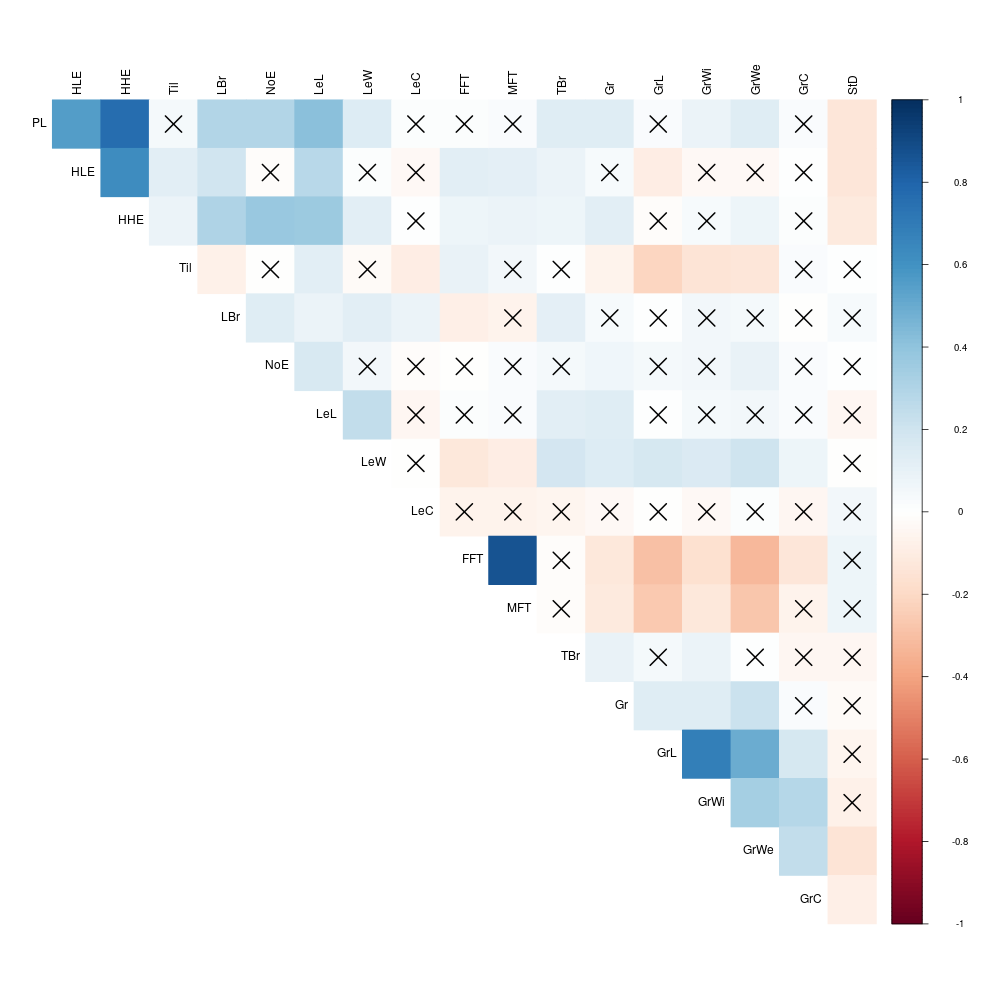

Supplement: S4 Fig — Pearson coefficient sign and magnitude for significant correlations between phenotypic traits after correction for experiment design (Model 2). X: correlations that are not significant. (TIF) [file pgen.1008512.s004.tif]

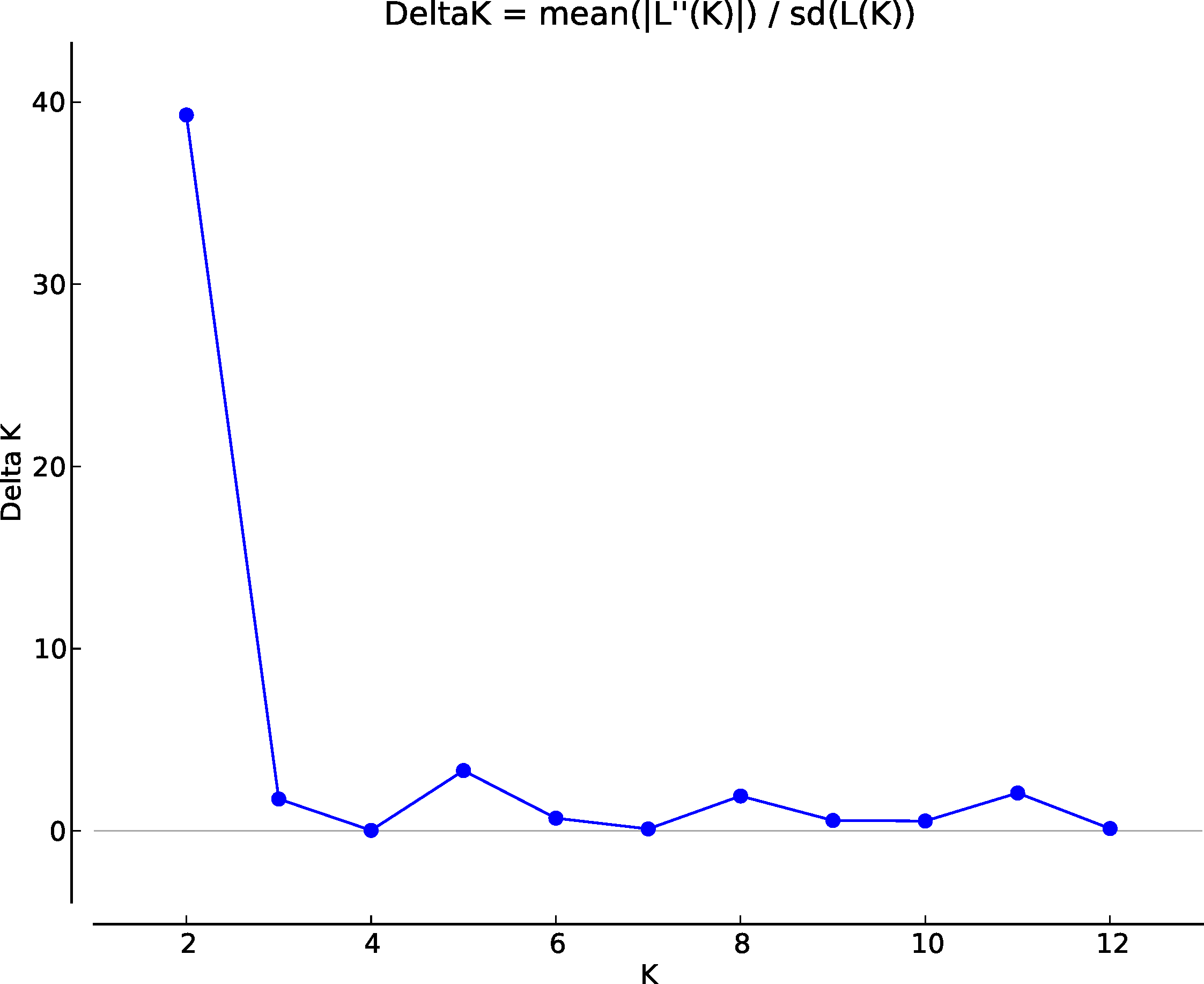

Supplement: S5 Fig — (TIF) [file pgen.1008512.s005.tif]

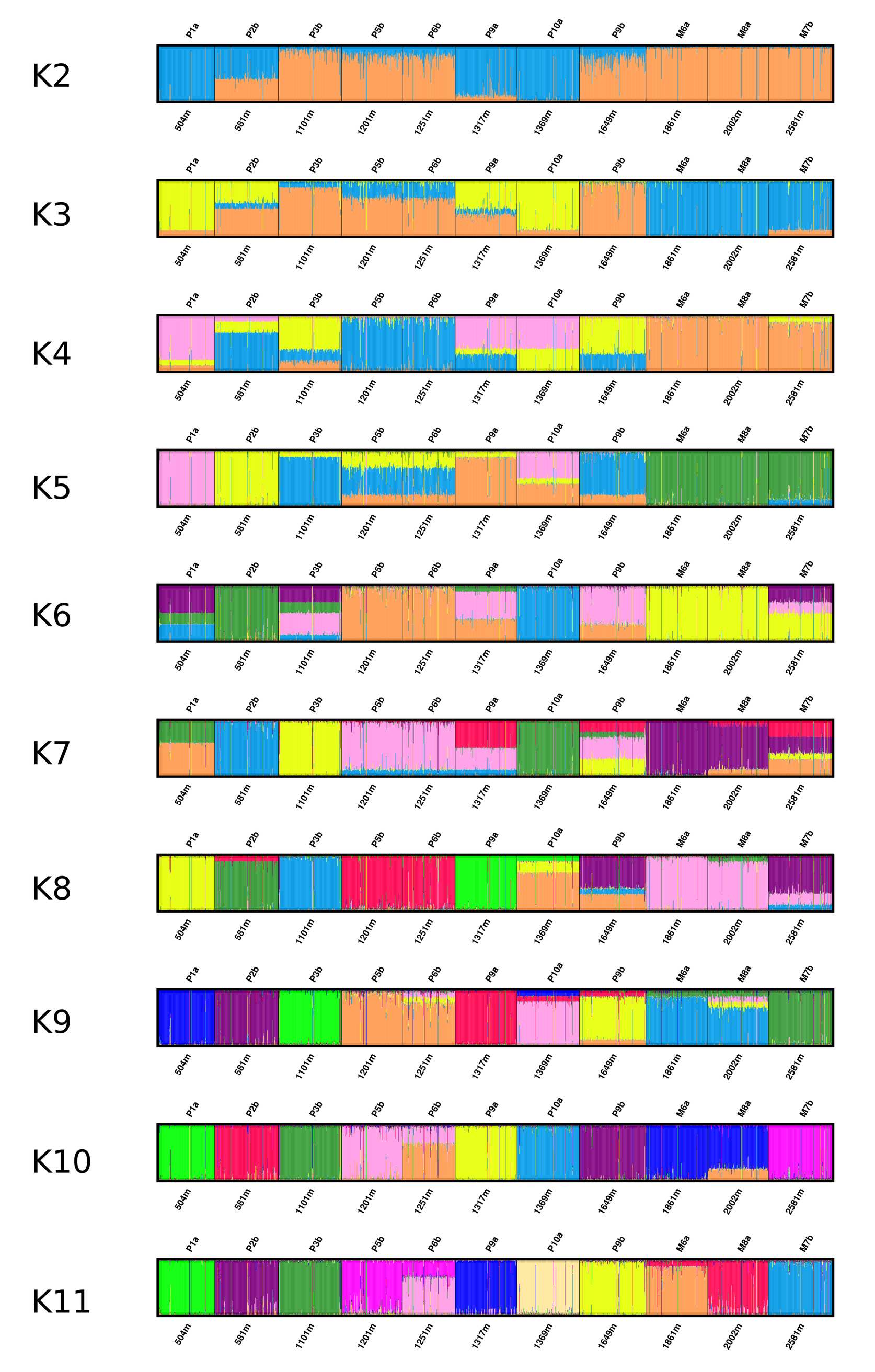

Supplement: S6 Fig — Genetic clustering was computed for K = 2 to K = 11. Vertical lines (individuals) are partitioned into coloured segments whose length represents the admixture proportions from the K clusters. (TIF) [file pgen.1008512.s006.tif]

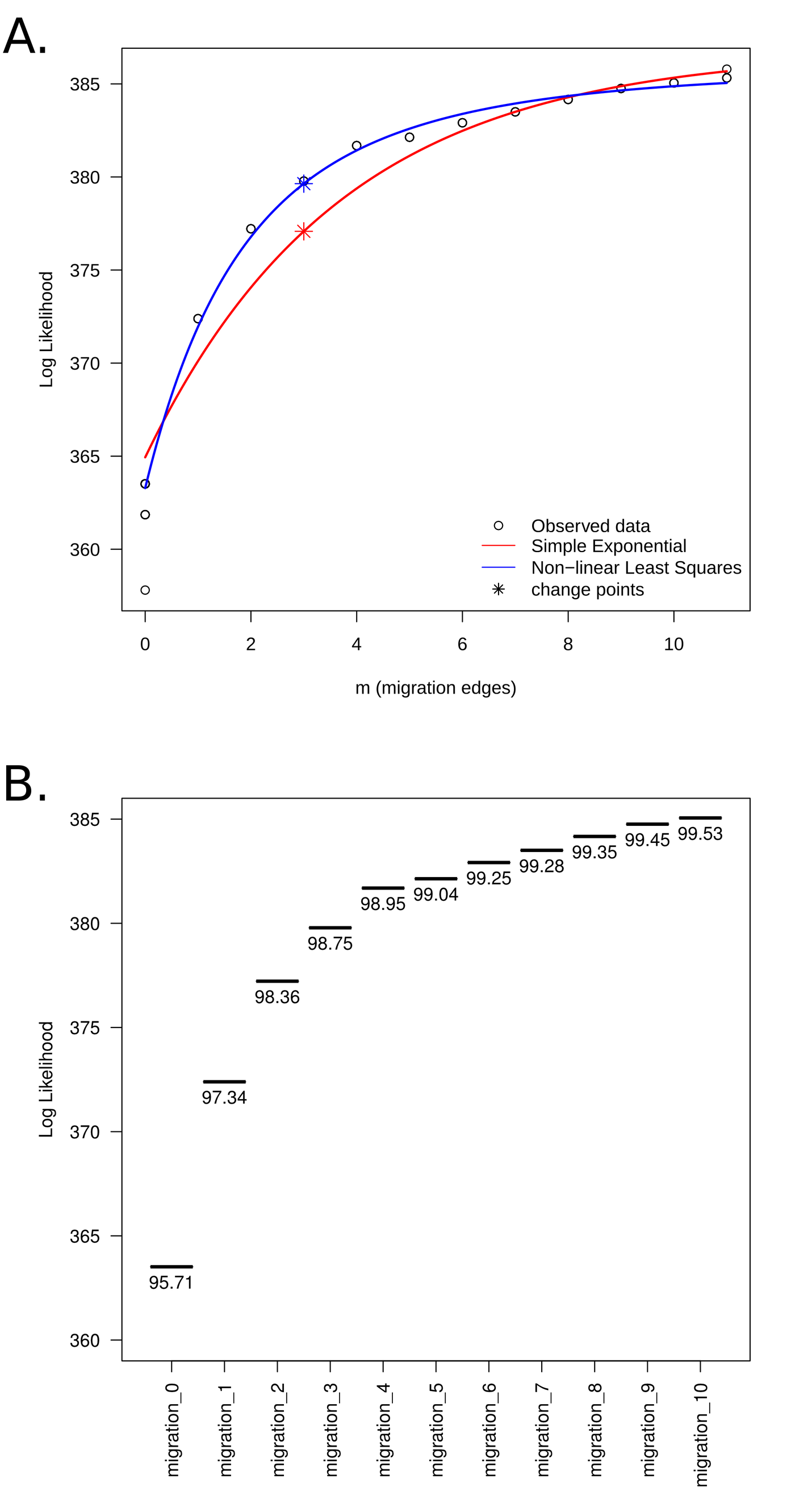

Supplement: S7 Fig — Observed Log likelihood values are plotted against the number of migration edges tested from 0 to 10, and two models are fitted to the data (A). Both the simple exponential and the non-linear least squares delivered an optimal value of 3 for the number of migration edges (change points). The model with 3 migration edges explained 98.75% of the variance, a substantial increase from the null model with no migration edge which is 95.7% (B). (TIF) [file pgen.1008512.s007.tif]

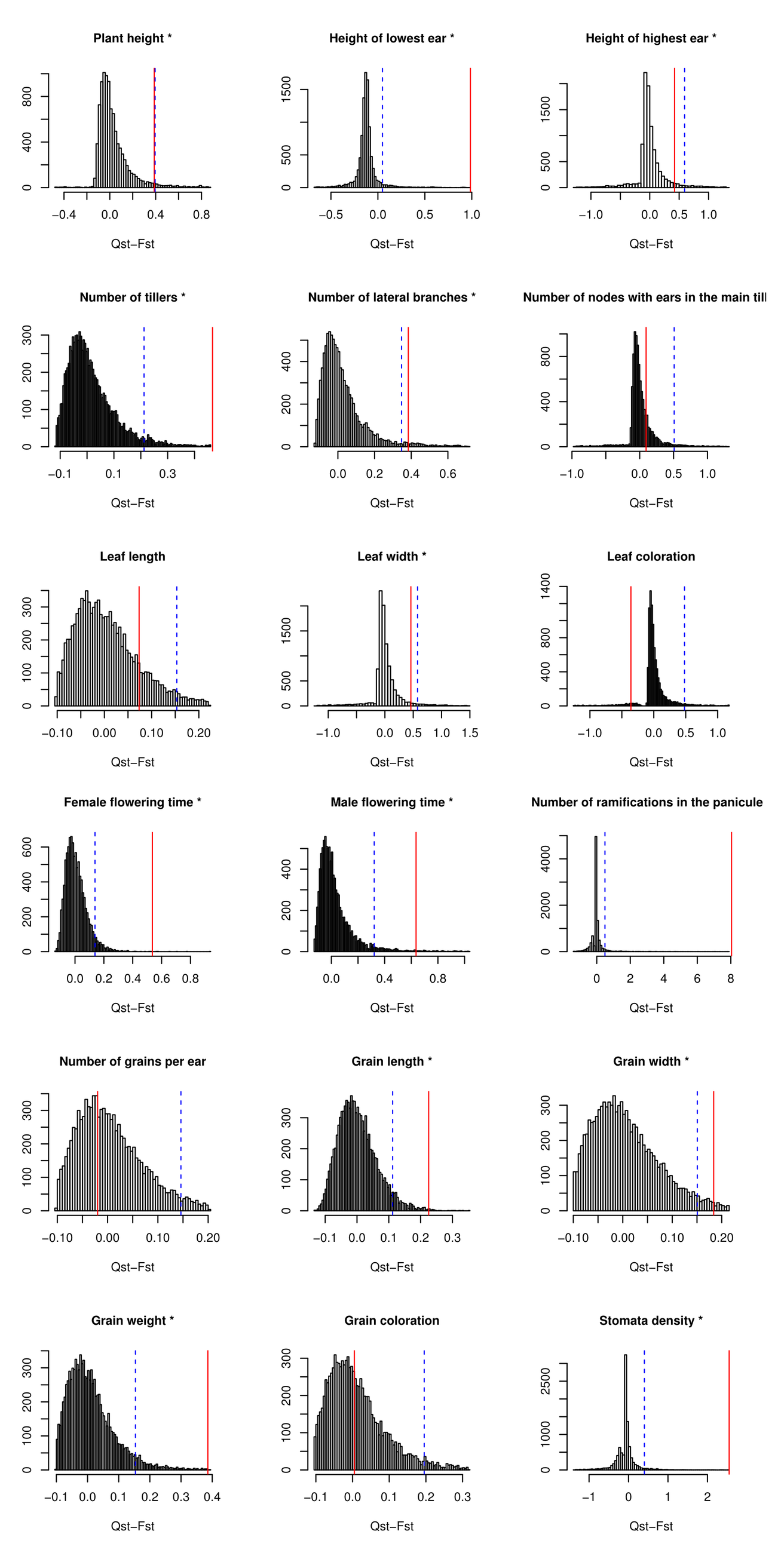

Supplement: S8 Fig — The dotted blue line indicates the 95% threshold of the simulated distributions and the red line refers to the observed difference. In this analysis, we considered as spatially-varying traits those for which the observed difference fell outside the 95% threshold. Note that Plant height was borderline significant. *: Set of traits detected by driftsel. (TIF) [file pgen.1008512.s008.tif]

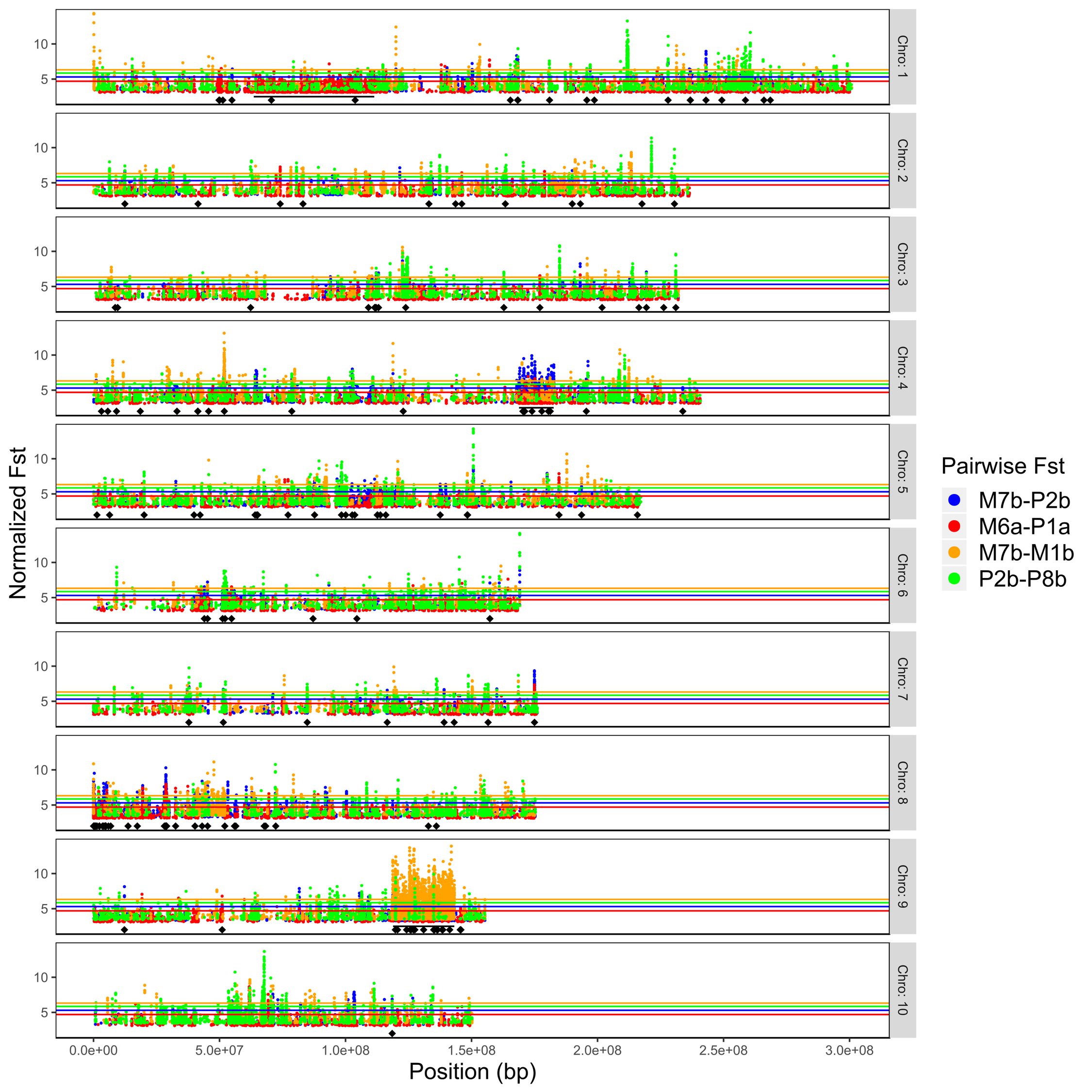

Supplement: S9 Fig — We computed 4 pairwise-FST values from 6 populations previously sequenced (S1 Table). Those include FST between lowland and highland populations of each gradient (P1a-M6a, P2b-M7b) as well as within subspecies on gradient b (P2b-P8b, M1b-M7b). FST values are averaged across sliding windows of 20 SNPs with a step of five SNPs (from top to bottom, chromosome 1 to 10) and normalized by subtracting the FST mean and dividing by the standard deviation across pairwise comparisons. Only the top 1% values are represented. The 1‰ thresholds for each pairwise comparisons are indicated by colored horizontal lines. Horizontal black bars indicate location of inversions on chromosome 1 (Inv1n), chromosome 4 (Inv4m) and chromosome 9 (Inv9d). The subset of 171 outlier SNPs analyzed in the present study is indicated with black diamond marks along the X axes. (TIF) [file pgen.1008512.s009.tif]

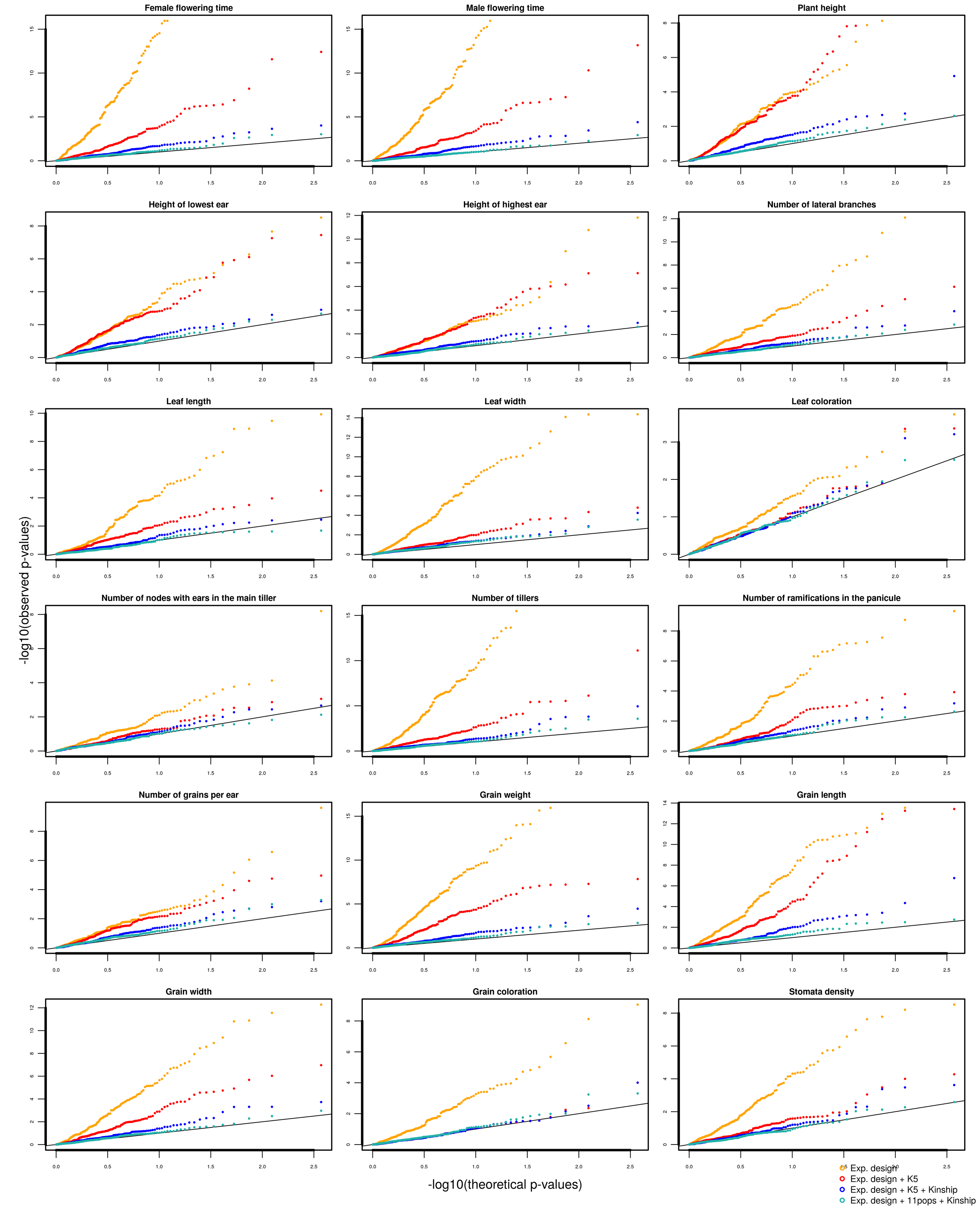

Supplement: S10 Fig — We employed three versions of the model 6 with correction for neither structure nor kinship, with correction for genetic structure (at K = 5), with correction for genetic structure (at K = 5 and with 11 populations) and kinship. (TIF) [file pgen.1008512.s010.tif]

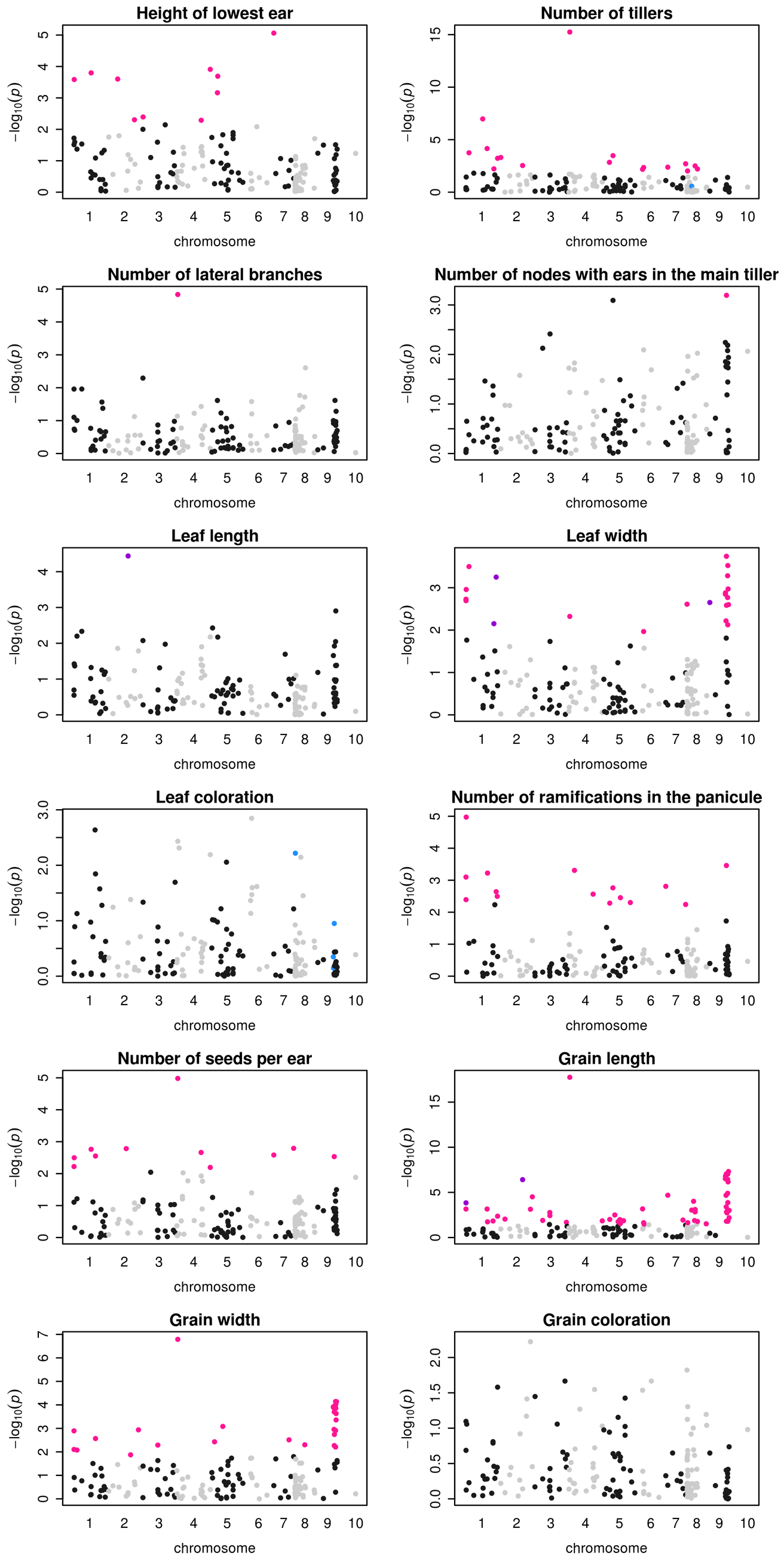

Supplement: S11 Fig — X-axis indicates the positions of outlier SNPs on chromosomes 1 to 10, black and gray colors alternating per chromosome. Plotted on the Y-axis are the negative Log10-transformed P values obtained for the K = 5 model. Significant associations (10% FDR) are indicated considering either a structure matrix at K = 5 (pink dots), for 11 populations (blue dots), or for both K = 5 and 11 populations models (purple dots). (TIF) [file pgen.1008512.s011.tif]

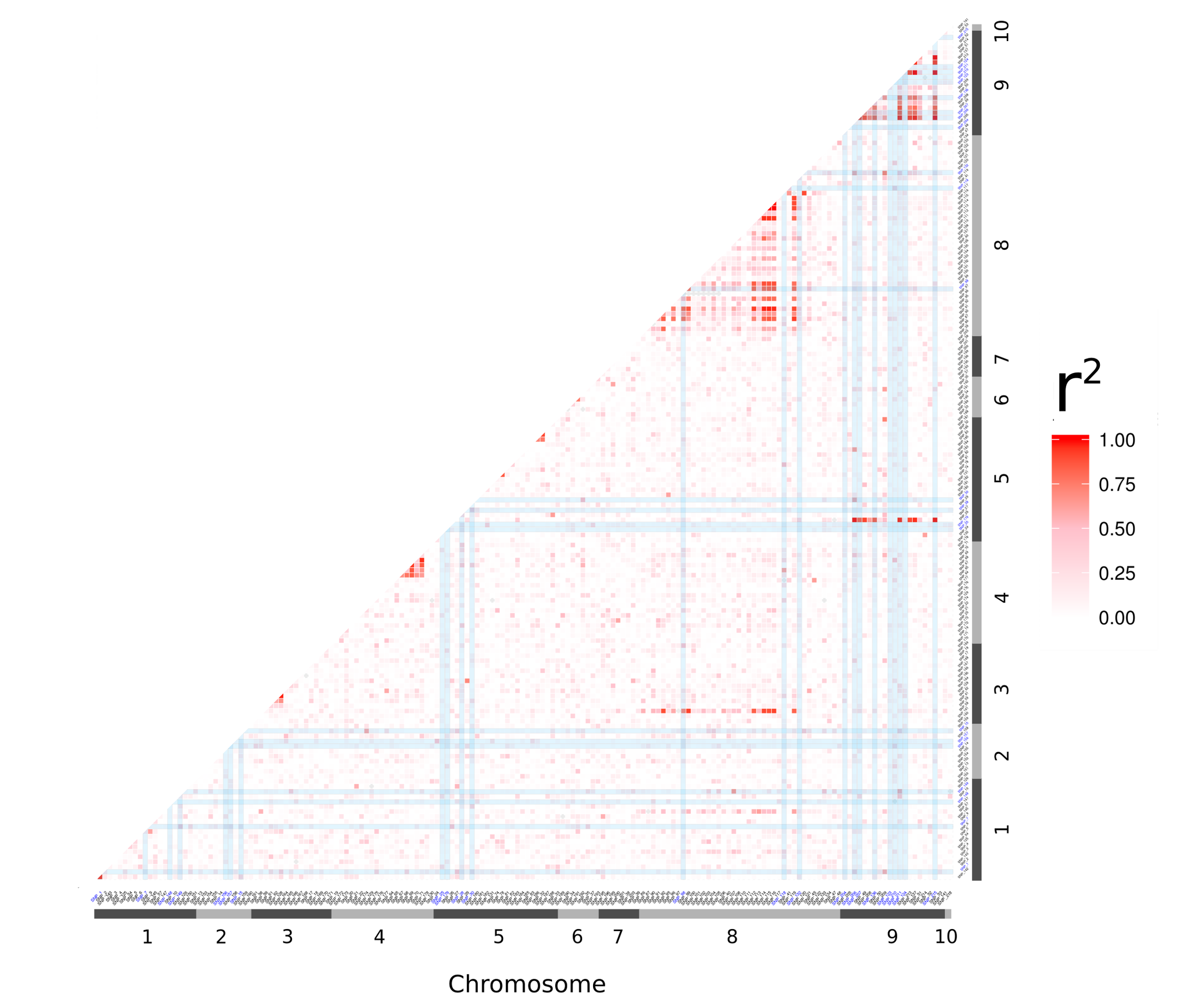

Supplement: S12 Fig — Pairwise LD between 171 SNPs was estimated using r2, and corrected for structure at K = 5 and kinship computed from 38 SSRs. Blue shaded bars show the 23 SNPs found to associate with at least one phenotype under the 11 populations structure correction. (TIF) [file pgen.1008512.s012.tif]
